# Unmasking the Area Postrema on MRI: Utility of 3D FLAIR, 3D-T2, and 3D-DIR Sequences in a Case–Control Study

**DOI:** 10.3390/jimaging11010016

**Published:** 2025-01-10

**Authors:** Javier Lara-García, Jessica Romo-Martínez, Jonathan Javier De-La-Cruz-Cisneros, Marco Antonio Olvera-Olvera, Luis Jesús Márquez-Bejarano

**Affiliations:** 1 Department of Radiology, Hospital de Especialidades del Centro Médico Nacional de Occidente, Mexican Social Security Institute IMSS, Guadalajara 44340, Mexico; jessica.romo1968@alumno.udg.mx (J.R.-M.); javierdelacruz@alumno.udg.mx (J.J.D.-L.-C.-C.); 2 Specialties Coordination, Centro Universitario de Ciencias de la Salud, University of Guadalajara, Guadalajara 44340, Mexico; 3 Hospital General de Zona No.21, Mexican Social Security Institute IMSS, León 37380, Mexico; ma.olveraolvera@ugto.mx; 4 Department of Physiology, Centro Universitario de Ciencias de la Salud, University of Guadalajara, Guadalajara 44340, Mexico

**Keywords:** area postrema, MRI, demyelinating diseases, multiple sclerosis, double inversion recovery

## Abstract

The area postrema (AP) is a key circumventricular organ involved in the regulation of autonomic functions. Accurate identification of the AP via MRI is essential in neuroimaging but it is challenging. This study evaluated 3D FSE Cube T2WI, 3D FSE Cube FLAIR, and 3D DIR sequences to improve AP detection in patients with and without multiple sclerosis (MS). A case–control study included 35 patients with MS and 35 with other non-demyelinating central nervous system diseases (ND-CNSD). MRI images were acquired employing 3D DIR, 3D FSE Cube FLAIR, and 3D FSE Cube T2WI sequences. The evaluation of AP was conducted using a 3-point scale. Statistical analysis was performed with the chi-square test used to assess group homogeneity and differences between sequences. No significant differences were found in the visualization of the AP between the MS and ND-CNSD groups across the sequences or planes. The AP was not visible in 27.6% of the 3D FSE Cube T2WI sequences, while it was visualized in 99% of the 3D FSE Cube FLAIR sequences and 100% of the 3D DIR sequences. The 3D DIR sequence showed superior performance in identifying the AP.

## 1. Introduction

The area postrema (AP) is located on the dorsomedial surface of the medulla oblongata, attached to the ventral angle of the floor of the fourth ventricle at the level of the obex [1]. It is a sensory circumventricular organ with afferent and efferent pathways [2] that, due to its close relationship with the nucleus of the solitary tract and the dorsal motor nucleus of the vagus, plays a critical role in the central regulation of autonomic functions [3]. Its most well-known function is serving as the trigger zone for nausea and vomiting [4], which is why the area postrema syndrome is defined as intractable episodes of nausea, vomiting, and/or hiccups in the context of a lesion at this level [5]. This syndrome is considered one of the key clinical features of neuromyelitis optica spectrum disorder (NMOSD) [6,7], being the primary finding in 10% of these patients and present in up to 30% at some point during the course of the disease [5,8].

Although lesions in the AP are not specific to NMOSD, and there are reports of patients with multiple sclerosis (MS) and area postrema syndrome [9,10], the presence of the clinical syndrome is highly specific for NMOSD [11]. However, there can be overlap in the radiological findings between these two diseases [12].

Therefore, accurate identification of this area through imaging methods is crucial for the diagnosis, differential diagnosis, and follow-up of various pathologies that may affect it [5,9,13,14,15].

In MRI studies, locating and accurately identifying the area postrema (AP) is challenging due to the small anatomical size of the structure and the lack of a reference sequence for its evaluation [4]. In recent years, various studies have attempted to determine the best sequence for its identification and localization, some of them using post-contrast GD-DTPA sequences [4,16,17]. However, there is no consensus or reference standard on the most sensitive sequence for detecting the AP in patients with and without demyelinating diseases of the central nervous system, nor is there clarity on whether the detection rate differs between these two populations [4].

This study analyzed the utility of the 3D Fast Spin Echo (FSE) Cube T2 Weighted Image (T2WI), 3D FSE Cube Fluid Attenuated Inversion Recovery (FLAIR), and 3D Double Inversion Recovery (DIR) sequences for the detection and localization of the AP in patients with and without MS, aiming to establish a reference technique for its evaluation and determine its normal standard in populations with and without inflammatory central nervous system pathologies.

## 2. Materials and Methods

This case–control study recruited 70 patients over the age of 18, 35 of whom had a previously established diagnosis of multiple sclerosis (MS) according to the 2017 McDonald criteria [18]. These patients were referred to the MRI service of a tertiary-level hospital in Guadalajara, Mexico. The control group included 35 patients with non-demyelinating central nervous system diseases (ND-CNSD), over 18 years of age who were referred to the MRI service of the same hospital. Exclusion criteria for both groups included a history of primary malignant neoplasia, metastatic disease to the central nervous system, congenital diseases of the central nervous system, infectious diseases of the central nervous system, and pregnant or breastfeeding patients. Images of insufficient quality were excluded from the sample. Informed consent was obtained from all participants.

The protocol was submitted to and approved by the local health research committee No. 1301, with approval number R-2023-1301-212.

### 2.1. Image Acquisition

A 3.0 T MR system (Discovery MR750w; General Electric) was used for imaging. For patients in the MS group, sequences were acquired according to the protocol recommended by the 2021 MAGNIMS-CMSC-NAIMS consensus [19]. In the group of patients with ND-CNSD, conventional sequences were acquired according to the imaging service protocols for diagnosis and/or follow-up, based on the suspected diagnosis. In both groups, 3D DIR, 3D FSE Cube FLAIR, and 3D FSE Cube T2WI sequences were obtained, with the characteristics detailed in Table 1, under the standardized parameters set by the manufacturer of the MRI equipment. These parameters are perfectly reproducible across different machines with the same magnetic field strength and achieve a balance between adequate image quality and short acquisition time.

The studies were anonymized at a dedicated workstation, a random identification number was assigned to each sequence, and the DICOM files of the images acquired with the sequences of interest were exported for subsequent analysis.

### 2.2. Image Analysis

A fourth-year resident in Diagnostic and Therapeutic Imaging and a board-certified radiologist from the Mexican Radiology and Imaging Council independently analyzed the images using a medical-grade monitor. In each sequence, the area postrema (AP) was evaluated as a hyperintense region on the dorsal surface of the lower medulla oblongata. Based on a 3-point ordinal scale, as described by Farges et al. [4], the degree of visibility was determined (0 = not visible, 1 = possibly visible, and 2 = clearly visible) for the sagittal plane and the coronal and axial reconstructions of the 3D FSE Cube FLAIR, 3D DIR, and 3D FSE Cube T2WI sequences. Concordant results between the two readers were accepted as valid. In case of disagreement, a certified neuroradiologist intervened and made the final determination regarding the degree of visibility.

### 2.3. Statistical Analysis

The statistical analysis was performed using SPSS version 27. The data were expressed and presented as measures of central tendency and dispersion. The homogeneity of the groups in terms of sex and age was determined using the chi-square test. Chi-square was also used as the inferential statistical method to compare the acquired sequences and the different planes of the sequences. A *p*-value of <0.05 was considered statistically significant.

## 3. Results

A total of 210 sequences were obtained (70 3D FSE Cube T2WI, 70 3D DIR, and 70 3D FSE Cube FLAIR), which were visualized in the axial, sagittal, and coronal planes, resulting in a total of 630 sequence-plane evaluations.

MRI protocols were performed on 35 patients diagnosed with MS (15 males and 20 females), with a mean age of 41 years (±11 years), and on 35 patients with ND-CNSD (17 males and 18 females), according to the selection criteria, with a mean age of 46 years (±17 years). No statistically significant differences were found between the groups in terms of age (*p* = 0.167) and sex (*p* = 0.631).

The disease duration reported by patients ranged from 1 to 47 years, with a mean of 11.4 ± 9 years. At the time of the study, 77% of the patients diagnosed with MS were under some form of treatment.

**Figure 1:**
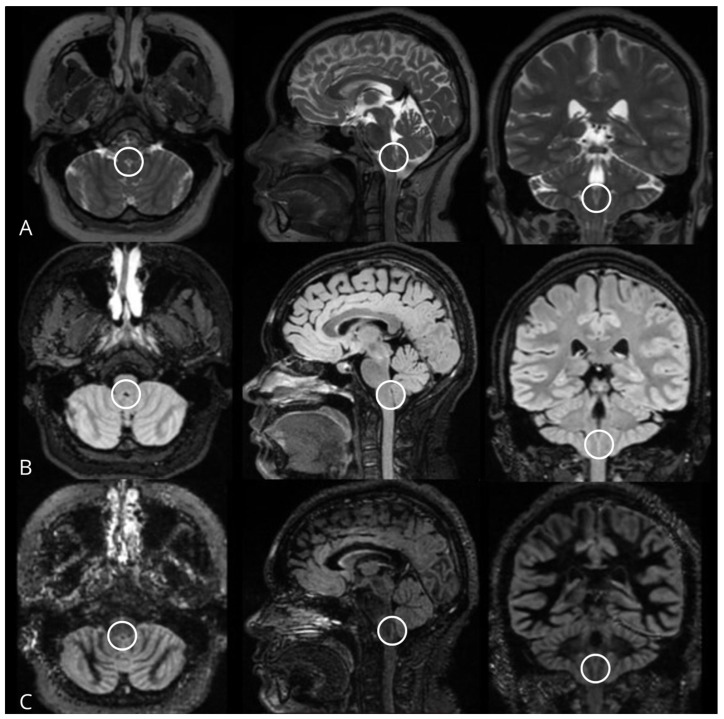
MRI of a control group patient. (**A**) 3D FSE Cube T2WI, (**B**) 3D FSE Cube FLAIR, (**C**) 3D DIR in axial (**left**), sagittal (**center**), and coronal (**right**) planes, demonstrating the area postrema (circles) as a linear hyperintense zone on the dorsomedial surface of the medulla oblongata, adhering to the ventral angle of the floor of the fourth ventricle at the level of the obex.

No statistically significant differences were found when comparing the degree of visualization in the different sequences between the group of patients with MS and the group with ND-CNSD (3D FSE Cube T2WI *p* = 0.286, 3D FSE Cube FLAIR *p* = 0.208, 3D DIR *p* = 0.307) (Figure 1) (Table 2).

Similarly, when comparing the degree of visualization in the different planes between the MS group and the ND-CNSD group, no statistically significant differences were found (axial *p* = 0.652, sagittal *p* = 0.327, coronal *p* = 0.462, overall *p* = 0.286).

**Figure 2:**
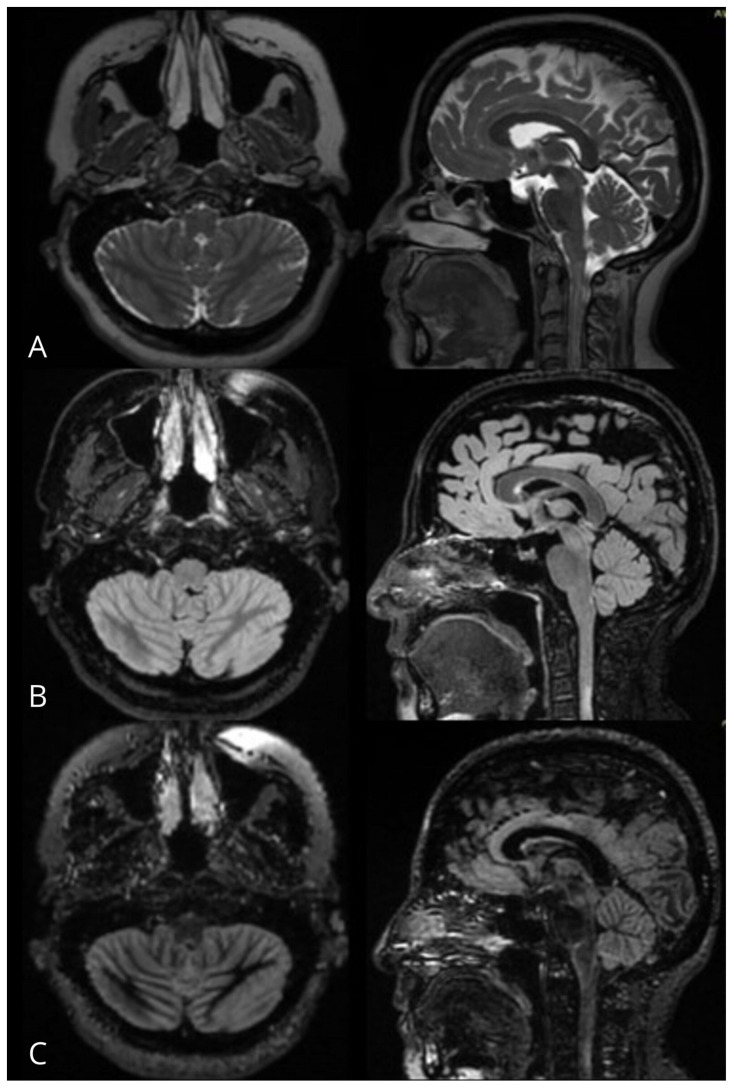
MRI of a patient with MS, in whom the area postrema cannot be identified in the 3D FSE Cube T2WI sequences (**A**) in axial and sagittal planes, but is visible in the axial and sagittal planes of 3D FSE Cube FLAIR (**B**) and 3D DIR (**C**) sequences.

In 27.6% of the 3D FSE Cube T2WI sequence-planes, it was not possible to visualize the AP, compared to 1% in the 3D FSE Cube FLAIR sequence. In contrast, the AP was visualized in 100% of the 3D DIR sequence-planes, at least at a “possible” level of visibility. Additionally, the 3D DIR sequence showed superior performance, with a greater number of sequence-planes displaying the AP as “clearly visible”, with statistically significant differences favoring the 3D DIR sequence (*p* < 0.001) (Figure 2) (Table 3).

## 4. Discussion

In our study, the AP was successfully visualized in both patients with MS and those with ND-CNSD, although there were variations in the proportion of visibility depending on the sequences and planes used. Notably, the visibility of the AP was consistent across both groups, regardless of the sequence employed. Although healthy patients were omitted in the study, the fact that it was visualized in the same proportion in both patients with MS and subjects with conditions that, according to the exclusion criteria, should not affect the area postrema suggests that the observed images are not due to pathological changes but rather to the intrinsic anatomical characteristics of the structure in these imaging techniques.

This could have significant clinical relevance, as the ability to accurately detect and evaluate this area is crucial for the diagnosis and monitoring of multiple neurological diseases, particularly NMOSD. In this condition, the clinical and imaging involvement of this structure represents one of the most specific manifestations of the disease and could, in many cases, aid in differentiating it from multiple sclerosis.

The consistency in AP visualization across both groups addresses a key question raised in the previous study by Farges et al. [4], while also providing a solid foundation for future research in healthy populations and on pathological changes in this region, aimed at establishing a baseline for AP visibility.

Another important aspect of this study was the demonstration of the capability of different MRI sequences to visualize the AP. The results showed that in 27.6% of the 3D FSE Cube T2WI sequence-planes, it was not possible to visualize the AP, compared to only 1% in the 3D FSE Cube FLAIR sequences. Notably, in 100% of the 3D DIR sequence-planes, the AP was visualized at least at the “possible” level.

Although previous studies, such as those by Horsburgh et al. [16] and Azuma et al. [17], attempted to identify various circumventricular organs through MRI, they relied on the post-contrast enhancement of these structures in different sequences. However, our findings align with those of Farges et al. [4], who focused specifically on the 3D DIR and 3D FSE Cube FLAIR sequences for the detection and localization of the AP in patients with MS. We believe this approach is more appropriate as it avoids the use of gadolinium, in line with current recommendations. Farges et al. [4] found that the 3D DIR sequence was superior to the 3D FSE Cube FLAIR in visualizing the AP, which is consistent with our results, where the 3D DIR sequence demonstrated significantly better capability in visualizing the AP compared to the 3D FSE Cube FLAIR and 3D FSE Cube T2WI sequences.

Additionally, the study confirmed the limited utility of the 3D FSE Cube T2WI sequences, which, despite having the same slice thickness (1.4 mm) as the 3D DIR sequences, did not allow for the identification of the AP. This may be due to what has been noted in previous research: without cerebrospinal fluid (CSF) suppression in the fourth ventricle, and given that the AP is normally hyperintense, its visualization becomes challenging. The inclusion of the 3D FSE Cube T2WI sequences in our study provides a comparative basis and broader context regarding the capabilities of the different sequences.

Although a total of 70 patients were evaluated, the sample size remains too small to generalize the findings to a broader population. Future studies in this area could benefit from a larger sample size to increase statistical power and the representativeness of the results.

The study design is cross-sectional, assessing lesion visibility at a single point in time. A longitudinal study that follows patients over time could provide valuable insights into lesion progression and the effectiveness of different MRI sequences in monitoring disease evolution.

It is acknowledged that the heterogeneity of conditions within the ND-CNSD control group can potentially introduce bias into the study’s results, particularly due to the absence of a true healthy control group and the lack of inclusion of patients with NMOSD for comparative analysis. Nevertheless, it is important to note the inherent ethical and methodological limitations of conducting imaging studies in a healthy population.

While this represents a limitation of the study, it also offers a valuable opportunity for future research. Addressing this gap, future studies could build on the insights gained here regarding the superiority of the 3D DIR sequence for visualizing the AP. Such research could focus on AP visualization in healthy subjects, using only 3D FLAIR and 3D DIR sequences, while excluding 3D T2WI sequences due to their confirmed limited utility in identifying the AP. This approach would allow for establishing streamlined protocols for addressing NMOSD, optimizing the use of MRI resources.

## 5. Conclusions

Our study demonstrated that the 3D DIR sequence is the most effective for visualizing the AP, achieving a 100% success rate, compared to the 3D FSE Cube FLAIR sequence (99%) and the 3D FSE Cube T2WI sequence (72.4%). Additionally, the consistency in AP visibility between patients with MS and those with ND-CNSD suggests that the observed differences are attributable to intrinsic anatomical characteristics rather than pathological variations. These findings have great potential to enhance the diagnosis and follow-up of neurological diseases that affect this anatomical structure.

## Figures and Tables

**Table 1:** Characteristics of sequences acquired.

Parameter	3D FSE Cube T2WI	3D FSE Cube FLAIR	3D DIR
Acquisition Plane	Oblique	Oblique	Oblique
Repetition Time (ms)	3000	6300	7000
Echo Time (ms)	Maximum	105	90
Echo Length	130	180	160
Inversion Time (ms)	N/A	1757	2889; 546
Bandwidth (Hz)	62.5	41.67	35.71
Slice Thickness (mm)	1.4	1	1.4
Number of Slices	256	256	512
Pixel Size	1.0 × 1.0	1.1 × 1.1	1.4 × 1.4
Acquisition Time (min)	02:46	06:03	04:44
Fat Saturation	No	Yes	Yes

Characteristics and parameters of 3D DIR, 3D FSE Cube FLAIR, and 3D FSE Cube T2WI sequences.

**Table 2:** Differences in visibility of the area postrema between cases and controls by sequence.

Sequence	Visibility	MS Cases	Controls
3D FSE Cube T2WI	Not visible	24 (22.9%) _a_	34 (32.4%) _a_
	Possible	58 (55.2%) _a_	49 (46.7%) _a_
	Visible	23 (21.9%) _a_	22 (21.0%) _a_
	Total	105 (100%)	105 (100%)
3D FSE Cube FLAIR	Not visible	2 (1.9%) _a_	0 (0%) _a_
	Possible	17 (16.2%) _a_	12 (11.4%) _a_
	Visible	86 (81.9%) _a_	93 (88.6%) _a_
	Total	105 (100%)	105 (100%)
3D DIR	Not visible	0 (0%) _a_	0 (0%) _a_
	Possible	6 (5.7%) _a_	3 (2.9%) _a_
	Visible	99 (94.3%) _a_	102 (97.1%) _a_
	Total	105 (100%)	105 (100%)

Each subscript letter (a) indicates subsets of categories whose column proportions do not differ significantly from each other at the *p* > 0.05 level.

**Table 3:** Differences in visibility of the area postrema among sequences by plane.

Plane	Visibility	3D FSE Cube T2WI	3D FSE Cube FLAIR	3D DIR
Axial	Not visible	17 (24.3%) _a_	0 (0.0%) _b_	0 (0.0%) _b_
	Possible	38 (54.3%) _a_	9 (12.9%) _b_	3 (4.3%) _b_
	Visible	15 (21.4%) _a_	61 (87.1%) _b_	67 (95.7%) _b_
	Total	70 (100%)	70 (100%)	70 (100%)
Sagittal	Not visible	21 (30.0%) _a_	1 (1.4%) _b_	0 (0.0%) _b_
	Possible	34 (48.6%) _a_	12 (17.1%) _b_	3 (4.3%) _c_
	Visible	15 (21.4%) _a_	57 (81.4%) _b_	67 (95.7%) _c_
	Total	70 (100%)	70 (100%)	70 (100%)
Coronal	Not visible	20 (28.6%) _a_	1 (1.4%) _b_	0 (0.0%) _b_
	Possible	35 (50.0%) _a_	8 (11.4%) _b_	3 (4.3%) _b_
	Visible	15 (21.4%) _a_	61 (87.1%) _b_	67 (95.7%) _b_
	Total	70 (100%)	70 (100%)	70 (100%)

Each subscript letter (a, b, c) indicates subsets of categories whose column proportions do not differ significantly from each other at the *p* > 0.05 level.

## Data Availability

Data from this research can be requested from the corresponding authors.
